# Supplementary material for: Treatment options and barriers to case management of neonatal pneumonia in India: a protocol for a scoping review
Source: BMJ Open. 2017 Sep 15;7(9):e017617. doi: 10.1136/bmjopen-2017-017617 (PMC5640122; doi:10.1136/bmjopen-2017-017617)
Supplement: Author's manuscript [file bmjopen-2017-017617.draft_revisions.pdf]

BMJ Open is committed to open peer review. As part of this commitment we make the peer review history of every article we publish publicly available.

When an article is published we post the peer reviewers' comments and the authors' responses online. We also post the versions of the paper that were used during peer review. These are the versions that the peer review comments apply to.

The versions of the paper that follow are the versions that were submitted during the peer review process. They are not the versions of record or the final published versions. They should not be cited or distributed as the published version of this manuscript.

BMJ Open is an open access journal and the full, final, typeset and author-corrected version of record of the manuscript is available on our site with no access controls, subscription charges or pay-per-view fees (http://bmjopen.bmj.com).

If you have any questions on BMJ Open's open peer review process please email editorial.bmjopen@bmj.com

BMJ Open

Treatment options and barriers to case management of neonatal pneumonia in India: a systematic review (Protocol)

Journal:	*BMJ Open*
Manuscript ID	bmjopen-2017-017617
Article Type:	Protocol
Date Submitted by the Author:	03-May-2017
Complete List of Authors:	Sreekumaran Nair, N; Manipal University, biostats<br>Lewis, Leslie; Manipal University Kasturba Medical College Manipal, paediatrics<br>Murthy, Shruti; Manipal University, Public Health Evidence South Asia (PHESA)<br>Lakiang, Theophilus; Manipal University<br>Godinho, Myron ; Manipal University, Public Health Evidence South Asia (PHESA)<br>Venkatesh , Bhumika; Manipal University
**Primary Subject Heading**:	Infectious diseases
Secondary Subject Heading:	Infectious diseases
Keywords:	Neonate, Pneumonia, Treatment, Case management, Barriers, Systematic Review

SCHOLARONE™
Manuscripts

For peer review only

Treatment options and Barriers to Case Management of Neonatal Pneumonia in India: A Systematic Review (Protocol)

N. Sreekumaran Nair[1], Leslie Edward Lewis[2], Shruti Murthy[3*], Myron Godinho[3**], Theophilus Lakiang[3***], Bhumika T. Venkatesh[4]

[1] Director, Public Health Evidence South Asia (PHESA), & Professor of Biostatistics and Head, Department of Statistics, Manipal University, Manipal. nsknairmanipal@gmail.com

[2] Professor and Head, Neonatology Unit, Department of Pediatrics, Kasturba Medical College, Manipal University, Manipal. leslie.lewis@manipal.edu

[3] Research Associate, Public Health Evidence South Asia (PHESA), Manipal University, Manipal;

*itzshruti@gmail.com

**myrongodinho@gmail.com

***theo.lakiang@gmail.com

[4]Assistant Professor, Public Health Evidence South Asia (PHESA), Manipal University, Manipal; meetbhumika123@gmail.com

Correspondence to:
Dr. N. Sreekumaran Nair
Director, Public Health Evidence South Asia (PHESA), Ground Floor, Manipal EDU Building, and
Professor of Biostatistics and Head, Department of Statistics,
Level 6, Health Science Library Building,
Manipal University,
Manipal-576104, Karnataka, India
Email address: nsknairmanipal@gmail.com
Telephone number: +91-8202922407

PROSPERO 2016:CRD42016045449

For peer review only

Treatment options and Barriers to Case Management of Neonatal Pneumonia in India: A Systematic Review (Protocol)

Abstract

Introduction: India contributes to the highest neonatal deaths globally. Case management is said to be the cornerstone of pneumonia control. Much of the published evidence focuses on children aged 1 to 59 months. This systematic review, thus, aims to identify the treatment options for and barriers to case management of neonatal pneumonia in India.

Methods and Analysis: This protocol is part of a series of three systematic reviews on neonatal pneumonia in India. Studies addressing treatment of or barriers to case management of neonatal pneumonia in Indian context, published in English in peer-reviewed and indexed journals will be eligible for inclusion. Electronic search will be conducted on nine databases. Hand searching and snowballing will be done for published and grey literature will be performed. Selection of studies will be done in title, abstract and full text stages. A narrative summary will be performed to summarize the details of evidence.

Ethics and dissemination: Since this is a review involving analysis of secondary data which is available in the public domain, and does not involve human participants, ethical approval was not required. The findings of the study will be shared with all stakeholders of this research. Knowledge dissemination workshops will be conducted with relevant stakeholders to ultimately transfer the evidence tailored to the stakeholder (e.g. policy briefs, publications, information booklets, etc).

Keywords: Neonate, Pneumonia, Treatment, Case management, Barriers, Systematic Review, India

For peer review only - http://bmjopen.bmj.com/site/about/guidelines.xhtml

Strengths and limitations of the study

- Most documented literature in pneumonia case management address post-neonatal age groups. First review to consolidate research on the treatment modalities, their implementation, and the barriers to the case management of neonatal pneumonia in India.

- A comprehensive search strategy was developed over 9 databases including relevant regional databases and grey literature published in any year.

- The review will narratively summarize published literature. No quality assessment of included studies is planned.

Introduction

Globally, 5.9 million children died in 2015, out of which 2.6 million were neonates.[1] India accounts for more neonatal deaths than any other country.[2] More than half of the child deaths from pneumonia occur during the newborn period.[3] In India, economic deprivation, impaired access to healthcare, harmful child-rearing practices, malnutrition and indoor air pollution are all major risk factors for pneumonia.[4 5] Neonatal pneumonia is particularly difficult to define and classify, as witnessed by differing definitions in different studies.[3 6]

In 2008, the World Health Organization (WHO)- United Nations Children's Fund (UNICEF) formulated the Global Action plan for the Prevention and control of Pneumonia (GAPP),[7] and in India, the Integrated Management of Neonatal & Childhood Illness (IMNCI) is now an important part of the national strategy to control childhood illness. Timely detection, effective case management, and prompt referral can reduce child morbidity and mortality due to pneumonia.[8] However, this is challenging in regions where co-morbid conditions (e.g. tuberculosis, malaria, AIDS) and antibiotic resistance prevail.[6] These difficulties are compounded by the clinical overlap of neonatal sepsis and pneumonia,[3] and obscured by conditions like hyaline membrane disease which mimic neonatal pneumonia, and impede detection in the absence of bacteriological confirmation.[5] Moreover, injudicious antibiotic therapy could lead to resistance or treatment failure.[9] A multitude of factors such as these pose special challenges to the initiation and maintenance of treatment, resulting in excessive morbidity and mortality.[8] However, most documented difficulties in pneumonia

For peer review only - http://bmjopen.bmj.com/site/about/guidelines.xhtml

case management address post-neonatal age groups, and fail to discuss neonatal treatment issues.

There is recognized need for consolidated research on the treatment modalities, their implementation, and the barriers to the case management of neonatal pneumonia in India. This evidence is required to inform the development of interventions, education and preventive strategies to combat this scourge of India's newborn. Thus this systematic review will attempt to synthesize evidence on different treatment options existing for neonatal pneumonia and the factors hindering effective case management of neonatal pneumonia in the Indian context.

The objectives of this systematic review are to identify the

1. Treatment options for neonatal pneumonia, and
2. Barriers to case management of neonatal pneumonia in the Indian context.

This protocol is part of a larger mixed-methods research project consisting of a qualitative study and a trilogy of systematic reviews on neonatal pneumonia in India addressing risk factors, management and predictors of mortality due to neonatal pneumonia in the Indian context.

Methods and analysis

This review will be conducted from August 2016 to October 2017.

Criteria for considering studies for this review

Types of studies

Inclusion criteria: Studies eligible for inclusion should have been conducted among neonates with pneumonia (or sepsis) in the Indian context, and their stakeholders. Eligible study designs will include editorials, case reports, case series, cross sectional studies, case control studies, cohort studies, intervention studies, qualitative studies, secondary data analysis, policy papers, guidelines, reports, fact sheets addressing treatment of or barriers to case management of neonatal pneumonia in Indian context. Studies have to be published in English language in indexed and peer-reviewed journals to be eligible for inclusion.

For peer review only - http://bmjopen.bmj.com/site/about/guidelines.xhtml

Exclusion criteria: The following studies will be excluded: all types of reviews, meta-analysis, conference papers and reports which do not report on treatment or barriers to case management of neonatal pneumonia in an Indian context.

Operational definitions: For the purpose of this review, treatment was operationally defined as ‘any specific or supportive treatment administered to a neonate with pneumonia’; case management was defined as ‘detection, investigation, treatment, referral, monitoring, support or follow up of pneumonia in a neonate either in the facility or community’[8]; and barrier to case management was defined as ‘any difficulty or obstacle during the case management of neonatal pneumonia’. Though we defined these terms in the beginning, our intention will also be to capture the definitions, where available, as reported by the authors and present them in the narrative synthesis.

Type of participants: Neonates with pneumonia in Indian context.

Outcomes of this review: Outcomes of this review will be (a) specific and supportive treatment of- and (b) barriers to case management of neonatal pneumonia in Indian context.

Search methods for identification of studies:

A comprehensive and relevant search strategy to identify all relevant studies will be developed by reviewing literature and discussion with subject experts and an information scientist. The search terms used and search strategies for PubMed have been provided in Tables 1 and 2.

Electronic searches: We will search PubMed, Ovid Medline, ProQuest, EMBASE, CINAHL, Web of Science, SCOPUS, WHOLIS and IndMED.

Hand searching: Hand searching will be conducted for reports/ guidelines/ journal volumes not included in electronic databases and conference proceedings to review the references and contact the authors for full text of identified literature.

Searching the grey literature: Potential sources of grey literature will include Shodhganga (INFLIBNET) and Government of India databases for reports, fact sheets and guidelines/ policies in the Indian context.

For peer review only

For peer review only - http://bmjopen.bmj.com/site/about/guidelines.xhtml

Reference lists: Snowballing will be conducted to screen the references of identified literature for potentially relevant studies.

Table 1: Search strategy for treatment options (PubMed):

Strategy: #1 AND #2 AND #3	
#1	(((Neonate* OR childhood OR neonatal* OR newborn* OR "young infant" OR child OR pediatric* OR "neonatal period" OR infant* OR "newborn infant")))
#2	((((((((((((((((((((((((((((((Pneumonia*) OR Pneumon*) OR "community acquired pneumonia") OR "congenital pneumonia") OR "hospital acquired pneumonia") OR "nosocomial pneumonia") OR "ventilator associated pneumonia") OR "early onset pneumonia") OR "late onset pneumonia") OR "infective pneumonia") OR "infectious pneumonia") OR "meconium aspiration syndrome") OR "meconium aspiration") OR "lipoid pneumonia") OR sepsis*) OR "acute respiratory infections") OR "early onset sepsis") OR "chemical pneumonia") OR "aspiration pneumonia") OR "late onset sepsis") OR infection*) OR "nosocomial infection") OR "early onset infection") OR "late onset infection") OR "acute lower respiratory infection") OR "hospital acquired infection") OR "congenital infection") OR "viral pneumonia") OR "gastro esophageal reflux disease") OR "cystic fibrosis")
#3	(((Treatment* OR Therap* OR "Patient care management" OR "Case management programs" OR "Home based neonatal care" OR "Case Management" OR "Clinical case management" OR "Community case management" OR "Integrated community case management" OR "Home based newborn care" OR "Case management models" OR Antibiotic* OR Ventilation* OR "Intensive care units" OR "Intensive care" OR "Neonatal intensive care units" OR "Special Newborn Care Units" OR "Injectable antibiotic" OR "oral antibiotic" OR "supportive therapy" OR "specific therapy" OR "specific treatment" OR "Supportive treatment"))))
Geographical filter: India<br>Language Filter: English	

Table 2: Search strategy for barriers to case management (PubMED)

Strategy: #1 AND #2 AND #3 AND #4	
#1	((((Neonate* OR childhood OR neonatal* OR newborn* OR "young infant" OR child OR pediatric* OR "neonatal period" OR infant* OR "newborn infant")))
#2	((((((((((((((((((((((((((((((Pneumonia*) OR Pneumon*) OR "community acquired pneumonia") OR "congenital pneumonia") OR "hospital acquired pneumonia") OR "nosocomial pneumonia") OR "ventilator associated pneumonia") OR "early onset pneumonia") OR "late onset pneumonia") OR "infective pneumonia") OR "infectious pneumonia") OR "meconium aspiration syndrome") OR "meconium aspiration") OR "lipoid pneumonia") OR sepsis*) OR "acute respiratory infections") OR "early onset sepsis") OR "chemical pneumonia") OR "aspiration pneumonia") OR "late onset sepsis") OR infection*) OR "nosocomial infection") OR "early onset infection") OR "late onset infection") OR "acute lower respiratory infection") OR "hospital acquired infection") OR

For peer review only - http://bmjopen.bmj.com/site/about/guidelines.xhtml

	"congenital infection") OR "viral pneumonia") OR "gastro esophageal reflux disease") OR "cystic fibrosis")
#3	((Treatment* OR Therap* OR "Patient care management" OR "Case management programs" OR "Home based neonatal care" OR "Case Management" OR "Clinical case management" OR "Community case management" OR "Integrated community case management" OR "Home based newborn care" OR "Case management models" OR Antibiotic* OR Ventilation* OR "Intensive care units" OR "Intensive care" OR "Neonatal intensive care units" OR "Special Newborn Care Units" OR "Injectable antibiotic" OR "oral antibiotic" OR "supportive therapy" OR "specific therapy" OR "specific treatment" OR "Supportive treatment"))
#4	(((Barriers* OR challenging OR challenge* OR obstacle* OR difficult* OR drawback OR problem* OR hurdle* OR hindrance* OR hinder* OR gap*)))))
Geographical filter: India<br>Language filter: English	

Data collection and management

The results (titles and/or abstracts) of the search will be managed using Endnote (v. x7). Study selection will be performed on Endnote (v. x7). Data will be extracted on Microsoft Excel 2007.

Selection of studies

Studies will be reviewed based on the exclusion and inclusion criteria by two authors (SM and TL) independently in three stages. Stage one or title screening will include assessment of each title for inclusion in the systematic review. If both authors reject a title, it will not be included in the review. Studies which are approved by either author will move to the second stage of appraisal. In the second or abstract screening stage will involve screening of abstracts of the titles selected in stage one for inclusion in the review. If both authors reject an abstract at this stage, it will not be included in the review. Studies which are approved by either author will move to the third and final stage of appraisal. Stage three, the full text screening stage, will comprise of screening the full text of the abstracts selected in stage two. Only those studies approved by both the authors at this stage will be included in the review. In the event of a study accepted by one author and rejected by another, a third author (MG) and a senior reviewer (SN or LL) will arbitrate and a consensus will be reached on whether to include the study or not.

Data extraction

For peer review only

The data extraction form was developed, using Microsoft Excel 2007, through an iterative process involving discussions and pilot testing. After a round of discussion among the authors, senior reviewers, subject and clinical experts, and statisticians, the form was pilot-tested on one study of each type to ensure that it adequately facilitated the collection of essential information required for the narrative synthesis. The key headings under which data extraction will be done include (1) Study Characteristics (2) Methodological characteristics (3) Treatment options and barriers to case management and (4) Other important information.

A final list of articles will be prepared for data extraction. This standardised, pre-tested data extraction form will be used independently by two authors (SM and TL) to extract data from the selected studies. Disagreements will be resolved in the presence of third (MG) and senior reviewer authors (SN and LL) by discussion and consensus. Any discrepancies regarding inclusion of the study in the review will be discussed with the team and advisory group, and a decision will be made regarding its inclusion in the review.

Dealing with missing data

In case of inadequacy, missing information, lack of clarity on information in methodology or if outcomes are missing, authors of the respective studies will be contacted in an attempt to obtain the required details. Despite this attempt, if the missing data retrieval on some aspects of the outcome (like clarity, inadequacy) is not possible, the study will be included in the narrative summary with a mention of the same.

Data Synthesis

A narrative summary will be performed to summarize the details of evidence. A discussion, where applicable, on study limitations that should be considered when interpreting the findings of the review will be included. The complete results of any analyses conducted, including the final search strategy, will be reported. No quality assessment of the included studies has been planned.

Quality Control and Reporting of the systematic review: A Preferred Reporting Items for Systematic reviews and Meta-analysis (PRISMA) chart will be created, to outline and

For peer review only - http://bmjopen.bmj.com/site/about/guidelines.xhtml

summarise this study selection process.[10] The findings of this systematic review will be reported in accordance with the PRISMA Guidelines.[10 11]

Ethics and dissemination:

Ethics: Since this is a review involving analysis of secondary data which is available in the public domain, and does not involve human participants, ethical approval was not required.

Dissemination: The findings of the study will be shared with all stakeholders of this research. Knowledge dissemination workshops will be conducted with relevant stakeholders to ultimately transfer the evidence tailored to the stakeholder (e.g. policy briefs, publications, information booklets, etc).

Acknowledgements

We would like to thank the following individuals for their continuous support and guidance during this process of protocol development: Dr. Manoj Das, Director Projects, The INCLEN Trust International, New Delhi; Dr Anju Sinha, Deputy Director General, Scientist 'E', Division of Child Health, Indian Council of Medical Research, New Delhi; Dr. K.K. Diwakar, Professor and Head, Department of Neonatology, Associate Dean, Malankara Orthodox Syrian Church Medical College, Kerala; Mrs. Ratheebhai V., Senior Librarian and Information Scientist, at Manipal School at Communication, Manipal University, Manipal; Dr. Ravinder M. Pandey, Professor and Head, Department of Biostatistics, All India Institute of Medical Sciences, New Delhi; Dr. B. Shantharam Baliga, Professor, Department of Paediatrics, Kasturba medical college, Mangalore, Karnataka, ; Dr. Shirish Darak, Senior researcher, PRAYAS, Pune, Maharashtra; Dr. Unnikrishnan B., Associate Dean and Professor, Department of Community Medicine, Kasturba Medical College, Mangalore. We also thank Public Health Evidence South Asia (PHESA) and Manipal University, Manipal for providing the necessary institutional and infrastructural support for the project. We would also like to thank The INCLEN Trust International, New Delhi, and The Bill and Melinda Gates Foundation for the financial support which made this project possible.

Contributorship statement

SN is the guarantor of the review. SN, BV AND LL conceived the research idea and reviewed the manuscript. SN and LL provided overall technical guidance. In addition, LL assisted in

For peer review only - http://bmjopen.bmj.com/site/about/guidelines.xhtml

For peer review only

developing search terms. SM, TL and MG designed the protocol, drafted the manuscript and developed and pilot tested the search strategies and data extraction form.

Funding: This project is supported by a grant from Bill and Melinda Gates Foundation (grant OPP1084307) to The INCLEN Trust International and sub-grant to Manipal University (subgrant INC2015GNT004). The views expressed through this project do not necessarily represent the views of Bill and Melinda Gates Foundation or The INCLEN Trust International or Manipal University.

Data sharing statement: All data supporting this study will be provided as supplementary material together with the manuscript of the study's final results.

Competing Interests: All authors have completed the ICMJE uniform disclosure form at www.icmje.org/coi_disclosure.pdf and declare: all authors had financial support (grants) from Bill and Melinda Gates Foundation (grant OPP1084307) to The INCLEN Trust International and sub-grant to Manipal University (subgrant INC2015GNT004)., during the conduct of the study and for the submitted work; no financial relationships with any organisations that might have an interest in the submitted work in the previous three years; no other relationships or activities that could appear to have influenced the submitted work.

References

1. UNICEF. Committing to Child Survival: A Promise Renewed, Progress Report 2015. 2015. *New York, USA: UNICEF* September 2015
2. Rudan I, Boschi-Pinto C, Biloglav Z, et al. Epidemiology and etiology of childhood pneumonia. *Bulletin of the World Health Organization* 2008;86(5):408-16B.
3. Reiterer F. Neonatal pneumonia. *Neonatal Bacterial Infection InTech* 2013
4. Ghimire M, Bhattacharya S, Narain J. Pneumonia in South-East Asia Region: Public health perspective. *The Indian journal of medical research* 2012;135(4):459.
5. Duke T. Neonatal pneumonia in developing countries. *Archives of Disease in Childhood-Fetal and Neonatal Edition* 2005;90(3):F211-FF19.
6. UNICEF. Integrated Global Action Plan for Prevention and Control of Pneumonia and Diarrhoea (GAPPD) 2013 [accessed 2016 20/1/2016]. Available from: http://www.who.int/
7. UNICEF, World Health Organization. Global action plan for prevention and control of pneumonia (GAPP). 2009
8. Graham SM, English M, Hazir T, et al. Challenges to improving case management of childhood pneumonia at health facilities in resource-limited settings. *Bulletin of the World Health Organization* 2008;86(5):349-55.
9. Engle WD, Jackson GL, Sendelbach DM, et al. Pneumonia in term neonates: laboratory studies and duration of antibiotic therapy. *Journal of Perinatology* 2003;23(5):372-77.

For peer review only - http://bmjopen.bmj.com/site/about/guidelines.xhtml

10. Liberati A, Altman DG, Tetzlaff J, et al. The PRISMA statement for reporting systematic reviews and meta-analyses of studies that evaluate health care interventions: explanation and elaboration. *Annals of internal medicine* 2009;151(4):W-65-W-94.
11. Moher D, Liberati A, Tetzlaff J, et al. Preferred reporting items for systematic reviews and meta-analyses: the PRISMA statement. *Annals of internal medicine* 2009;151(4):264-69.

For peer review only

For peer review only - http://bmjopen.bmj.com/site/about/guidelines.xhtml

PRISMA-P (Preferred Reporting Items for Systematic review and Meta-Analysis Protocols) 2015 checklist: recommended items to address in a systematic review protocol*

Section and topic	Item No	Checklist item	Compliance
**ADMINISTRATIVE INFORMATION**			
Title:			
Identification	1a	Identify the report as a protocol of a systematic review	Pg 1& 2
Update	1b	If the protocol is for an update of a previous systematic review, identify as such	Not applicable
Registration	2	If registered, provide the name of the registry (such as PROSPERO) and registration number	Pg 1
Authors:			
Contact	3a	Provide name, institutional affiliation, e-mail address of all protocol authors; provide physical mailing address of corresponding author	Pg 1
Contributions	3b	Describe contributions of protocol authors and identify the guarantor of the review	Pg 9
Amendments	4	If the protocol represents an amendment of a previously completed or published protocol, identify as such and list changes; otherwise, state plan for documenting important protocol amendments	Not applicable
Support:			
Sources	5a	Indicate sources of financial or other support for the review	Pg 10
Sponsor	5b	Provide name for the review funder and/or sponsor	Pg 10
Role of sponsor or funder	5c	Describe roles of funder(s), sponsor(s), and/or institution(s), if any, in developing the protocol	Pg 10
**INTRODUCTION**			
Rationale	6	Describe the rationale for the review in the context of what is already known	Pg 3-4
Objectives	7	Provide an explicit statement of the question(s) the review will address with reference to participants, interventions, comparators, and outcomes (PICO)	Pg 4 Modified accordingly for review of non-interventional studies
**METHODS**			
Eligibility criteria	8	Specify the study characteristics (such as PICO, study design, setting, time frame) and report characteristics (such as years considered, language, publication status) to be used as criteria for eligibility for the review	Pg 4-5 Modified accordingly for

For peer review only

			review of non-interventional studies
Information sources	9	Describe all intended information sources (such as electronic databases, contact with study authors, trial registers or other grey literature sources) with planned dates of coverage	Pg 5-6
Search strategy	10	Present draft of search strategy to be used for at least one electronic database, including planned limits, such that it could be repeated	Pg 6-7
Study records:			
Data management	11a	Describe the mechanism(s) that will be used to manage records and data throughout the review	Pg 7
Selection process	11b	State the process that will be used for selecting studies (such as two independent reviewers) through each phase of the review (that is, screening, eligibility and inclusion in meta-analysis)	Pg 7
Data collection process	11c	Describe planned method of extracting data from reports (such as piloting forms, done independently, in duplicate), any processes for obtaining and confirming data from investigators	Pg 8
Data items	12	List and define all variables for which data will be sought (such as PICO items, funding sources), any pre-planned data assumptions and simplifications	Pg 8
Outcomes and prioritization	13	List and define all outcomes for which data will be sought, including prioritization of main and additional outcomes, with rationale	Pg 8
Risk of bias in individual studies	14	Describe anticipated methods for assessing risk of bias of individual studies, including whether this will be done at the outcome or study level, or both; state how this information will be used in data synthesis	Pg 8
Data synthesis	15a	Describe criteria under which study data will be quantitatively synthesised	Pg 8
	15b	If data are appropriate for quantitative synthesis, describe planned summary measures, methods of handling data and methods of combining data from studies, including any planned exploration of consistency (such as I^2, Kendall's τ)	No quantitative synthesis planned
	15c	Describe any proposed additional analyses (such as sensitivity or subgroup analyses, meta-regression)	
	15d	If quantitative synthesis is not appropriate, describe the type of summary planned	Pg 8
Meta-bias(es)	16	Specify any planned assessment of meta-bias(es) (such as publication bias across studies, selective reporting within studies)	NIL
Confidence in cumulative evidence	17	Describe how the strength of the body of evidence will be assessed (such as GRADE)	None planned

*** It is strongly recommended that this checklist be read in conjunction with the PRISMA-P Explanation and Elaboration (cite when available) for important clarification on the items. Amendments to a review protocol should be tracked and dated.** **The copyright for PRISMA-P (including checklist) is held by the PRISMA-P Group and is distributed under a Creative Commons Attribution Licence 4.0.**

From: Shamseer L, Moher D, Clarke M, Ghersi D, Liberati A, Petticrew M, Shekelle P, Stewart L, PRISMA-P Group. Preferred reporting items for systematic review and meta-analysis protocols (PRISMA-P) 2015: elaboration and explanation. BMJ. 2015 Jan 2;349(jan02 1):g7647.

For peer review only

For peer review only

BMJ Open

Treatment options and Barriers to Case Management of Neonatal Pneumonia in India: a protocol for a scoping review

Journal:	*BMJ Open*
Manuscript ID	bmjopen-2017-017617.R1
Article Type:	Protocol
Date Submitted by the Author:	21-Jun-2017
Complete List of Authors:	Sreekumaran Nair, N; Manipal University, biostats<br>Lewis, Leslie; Manipal University Kasturba Medical College Manipal, paediatrics<br>Murthy, Shruti; Manipal University, Public Health Evidence South Asia (PHESA)<br>Godinho, Myron ; Manipal University, Public Health Evidence South Asia (PHESA)<br>Lakiang, Theophilus; Manipal University<br>Venkatesh , Bhumika; Manipal University
**Primary Subject Heading**:	Infectious diseases
Secondary Subject Heading:	Infectious diseases, Paediatrics
Keywords:	Neonate, Pneumonia, Treatment, Case management, Barriers, scoping review

SCHOLARONE™ Manuscripts

For peer review only

Treatment options and Barriers to Case Management of Neonatal Pneumonia in India: a protocol for a scoping review

N. Sreekumaran Nair[1], Leslie Edward Lewis[2], Shruti Murthy [3*], Myron Godinho [3**], Theophilus Lakiang[3***], Bhumika T. Venkatesh[4]

[1] Director, Public Health Evidence South Asia (PHESA), & Professor of Biostatistics and Head, Department of Statistics, Manipal University, Manipal. sree.nair@manipal.edu, nsknairmanipal@gmail.com

[2] Professor and Head, Neonatology Unit, Department of Pediatrics, Kasturba Medical College, Manipal University, Manipal. leslie.lewis@manipal.edu

[3] Research Associate, Public Health Evidence South Asia (PHESA), Manipal University, Manipal;

*itzshruti@gmail.com

**myrongodinho@gmail.com

***theo.lakiang@gmail.com

[4]Assistant Professor, Public Health Evidence South Asia (PHESA), Manipal University, Manipal; meetbhumika123@gmail.com

Correspondence to:
Dr. N. Sreekumaran Nair
Director, Public Health Evidence South Asia (PHESA), Ground Floor, Manipal EDU Building, and
Professor of Biostatistics and Head, Department of Statistics,
Level 6, Health Science Library Building,
Manipal University,
Manipal-576104, Karnataka, India
Email address: sree.nair@manipal.edu, nsknairmanipal@gmail.com
Telephone number: +91-8202922407

PROSPERO 2016:CRD42016045449

For peer review only

For peer review only - http://bmjopen.bmj.com/site/about/guidelines.xhtml

Treatment options and Barriers to Case Management of Neonatal Pneumonia in India: a protocol for a scoping review

Abstract

Introduction: India contributes to the highest neonatal deaths globally. Case management is said to be the cornerstone of pneumonia control. Much of the published evidence focuses on children aged 1 to 59 months. This scoping review, thus, aims to identify the treatment options for and barriers to case management of neonatal pneumonia in India.

Methods and Analysis: This protocol is part of a series of three reviews on neonatal pneumonia in India. Studies addressing treatment of or barriers to case management of neonatal pneumonia in Indian context, published in English in peer-reviewed and indexed journals will be eligible for inclusion. Electronic search will be conducted on nine databases. Hand searching and snowballing will be done for published and grey literature will be performed. Selection of studies will be done in title, abstract and full text stages. A narrative summary will be performed to summarize the details of evidence.

Ethics and dissemination: Since this is a review involving analysis of secondary data which is available in the public domain, and does not involve human participants, ethical approval was not required. The findings of the study will be shared with all stakeholders of this research. Knowledge dissemination workshops will be conducted with relevant stakeholders to ultimately transfer the evidence tailored to the stakeholder (e.g. policy briefs, publications, information booklets, etc).

Keywords: Neonate, Pneumonia, Treatment, Case management, Barriers, Scoping Review, India

For peer review only

Strengths and limitations of the study

- Most documented literature in pneumonia case management address post-neonatal age groups. First review to consolidate research on the treatment modalities, their implementation, and the barriers to the case management of neonatal pneumonia in India.

- A comprehensive search strategy was developed over 9 databases including relevant regional databases and grey literature published in any year.

- The review will narratively summarize published literature. No quality assessment of included studies is planned.

Introduction

Globally, 5.9 million children died in 2015, out of which 2.6 million were neonates.[1] India accounts for more neonatal deaths than any other country.[2] More than half of the child deaths from pneumonia occur during the newborn period.[3] In India, economic deprivation, impaired access to healthcare, harmful child-rearing practices, malnutrition and indoor air pollution are all major risk factors for pneumonia.[4 5] Neonatal pneumonia is particularly difficult to define and classify, as witnessed by differing definitions in different studies.[3 6]

In 2008, the World Health Organization (WHO)- United Nations Children's Fund (UNICEF) formulated the Global Action plan for the Prevention and control of Pneumonia (GAPP),[7] and in India, the Integrated Management of Neonatal & Childhood Illness (IMNCI) is now an important part of the national strategy to control childhood illness. Timely detection, effective case management, and prompt referral can reduce child morbidity and mortality due to pneumonia.[8] However, this is challenging in regions where co-morbid conditions (e.g. tuberculosis, malaria, AIDS) and antibiotic resistance prevail.[6] These difficulties are compounded by the clinical overlap of neonatal sepsis and pneumonia,[3] and obscured by conditions like hyaline membrane disease which mimic neonatal pneumonia, and impede detection in the absence of bacteriological confirmation.[5] Moreover, injudicious antibiotic therapy could lead to resistance or treatment failure.[9] A multitude of factors such as these pose special challenges to the initiation and maintenance of treatment, resulting in excessive morbidity and mortality.[8] However, most documented difficulties in pneumonia

For peer review only - http://bmjopen.bmj.com/site/about/guidelines.xhtml

case management address post-neonatal age groups, and fail to discuss neonatal treatment issues.

There is recognized need for consolidated research on the treatment modalities, their implementation, and the barriers to the case management of neonatal pneumonia in India. This evidence is required to inform the development of interventions, education and preventive strategies to combat this scourge of India's newborn. Thus this scoping review will attempt to synthesize evidence on different treatment options existing for neonatal pneumonia and the factors hindering effective case management of neonatal pneumonia in the Indian context.

The objectives of this scoping review are to identify the

1. Treatment options for neonatal pneumonia, and
2. Barriers to case management of neonatal pneumonia in the Indian context.

This protocol is part of a larger mixed-methods research project consisting of a qualitative study and a trilogy of two systematic and one scoping reviews on neonatal pneumonia in India addressing risk factors, management and predictors of mortality due to neonatal pneumonia in the Indian context.

Methods and analysis

This review will be conducted from August 2016 to October 2017.

Criteria for considering studies for this review

Types of studies

Inclusion criteria: Studies eligible for inclusion should have been conducted among neonates with pneumonia (or sepsis) in the Indian context, and their stakeholders. Primary studies, (of any study design including editorials, case reports, case series, cross-sectional studies, case control studies, cohort studies, intervention studies and qualitative studies), policy papers, guidelines, reports, and fact sheets, addressing treatment of or barriers to case management of neonatal pneumonia in Indian context were eligible to be included in the review. Studies have to be published in English language in indexed and peer-reviewed journals to be eligible for inclusion.

For peer review only - http://bmjopen.bmj.com/site/about/guidelines.xhtml

Exclusion criteria: The following studies will be excluded: all types of reviews, meta-analysis, conference papers and reports which do not report on treatment or barriers to case management of neonatal pneumonia in an Indian context.

Operational definitions: For the purpose of this review, treatment was operationally defined as ‘any specific or supportive treatment administered to a neonate with pneumonia’; case management was defined as ‘detection, investigation, treatment, referral, monitoring, support or follow up of pneumonia in a neonate either in the facility or community’[8]; and barrier to case management was defined as ‘any difficulty or obstacle during the case management of neonatal pneumonia’. Though we defined these terms in the beginning, our intention will also be to capture the definitions, where available, as reported by the authors and present them in the narrative synthesis.

Type of participants: Neonates with pneumonia in Indian context.

Outcomes of this review: Outcomes of this review will be (a) specific and supportive treatment of- and (b) barriers to case management of neonatal pneumonia in Indian context.

Search methods for identification of studies:

A comprehensive and relevant search strategy to identify all relevant studies will be developed by reviewing literature and discussion with subject experts and an information scientist. The search terms used and search strategies for PubMed have been provided in Tables 1 and 2.

Electronic searches: We will search PubMed, Ovid Medline, ProQuest, EMBASE, CINAHL, Web of Science, SCOPUS, WHOLIS and IndMED.

Hand searching: Hand searching will be conducted for reports/ guidelines/ journal volumes not included in electronic databases and conference proceedings to review the references and contact the authors for full text of identified literature.

Searching the grey literature: Potential sources of grey literature will include Shodhganga (INFLIBNET) and Government of India databases for reports, fact sheets and guidelines/ policies in the Indian context.

For peer review only - http://bmjopen.bmj.com/site/about/guidelines.xhtml

Reference lists: Snowballing will be conducted to screen the references of identified literature for potentially relevant studies.

Table 1: Search strategy for treatment options (PubMed):

Strategy: #1 AND #2 AND #3	
#1	(((Neonate* OR childhood OR neonatal* OR newborn* OR "young infant" OR child OR paediatric OR pediatric* OR "neonatal period" OR infant* OR "newborn infant")))
#2	((((((((((((((((((((((((((((((Pneumonia*) OR Pneumon*) OR "community acquired pneumonia") OR "congenital pneumonia") OR "hospital acquired pneumonia") OR "nosocomial pneumonia") OR "ventilator associated pneumonia") OR "early onset pneumonia") OR "late onset pneumonia") OR "infective pneumonia") OR "infectious pneumonia") OR "meconium aspiration syndrome") OR "meconium aspiration") OR "lipoid pneumonia") OR sepsis*) OR "acute respiratory infections") OR "early onset sepsis") OR "chemical pneumonia") OR "aspiration pneumonia") OR "late onset sepsis") OR infection*) OR "nosocomial infection") OR "early onset infection") OR "late onset infection") OR "acute lower respiratory infection") OR "hospital acquired infection") OR "congenital infection") OR "viral pneumonia") OR "gastro esophageal reflux disease") OR "cystic fibrosis")
#3	(((Treatment* OR Therap* OR "Patient care management" OR "Case management programs" OR "Home based neonatal care" OR "Case Management" OR "Clinical case management" OR "Community case management" OR "Integrated community case management" OR "Home based newborn care" OR "Case management models" OR Antibiotic* OR Ventilation* OR "Intensive care units" OR "Intensive care" OR "Neonatal intensive care units" OR "Special Newborn Care Units" OR "Injectable antibiotic" OR "oral antibiotic" OR "supportive therapy" OR "specific therapy" OR "specific treatment" OR "Supportive treatment"))))
Geographical filter: India<br>Language Filter: English	

Table 2: Search strategy for barriers to case management (PubMED)

Strategy: #1 AND #2 AND #3 AND #4	
#1	((((Neonate* OR childhood OR neonatal* OR newborn* OR "young infant" OR child OR paediatric OR pediatric* OR "neonatal period" OR infant* OR "newborn infant")))
#2	((((((((((((((((((((((((((((((Pneumonia*) OR Pneumon*) OR "community acquired pneumonia") OR "congenital pneumonia") OR "hospital acquired pneumonia") OR "nosocomial pneumonia") OR "ventilator associated pneumonia") OR "early onset pneumonia") OR "late onset pneumonia") OR "infective pneumonia") OR "infectious pneumonia") OR "meconium aspiration syndrome") OR "meconium aspiration") OR "lipoid pneumonia") OR sepsis*) OR "acute respiratory infections") OR "early onset sepsis") OR "chemical pneumonia") OR "aspiration pneumonia") OR "late onset sepsis") OR infection*) OR "nosocomial infection") OR "early onset infection") OR "late onset infection") OR "acute lower respiratory infection") OR "hospital acquired infection") OR

For peer review only

For peer review only - http://bmjopen.bmj.com/site/about/guidelines.xhtml

	"congenital infection") OR "viral pneumonia") OR "gastro esophageal reflux disease") OR "cystic fibrosis")
#3	((Treatment* OR Therap* OR "Patient care management" OR "Case management programs" OR "Home based neonatal care" OR "Case Management" OR "Clinical case management" OR "Community case management" OR "Integrated community case management" OR "Home based newborn care" OR "Case management models" OR Antibiotic* OR Ventilation* OR "Intensive care units" OR "Intensive care" OR "Neonatal intensive care units" OR "Special Newborn Care Units" OR "Injectable antibiotic" OR "oral antibiotic" OR "supportive therapy" OR "specific therapy" OR "specific treatment" OR "Supportive treatment"))
#4	(((Barriers* OR challenging OR challenge* OR obstacle* OR difficult* OR drawback OR problem* OR hurdle* OR hindrance* OR hinder* OR gap* OR cost* OR utilization OR satisfaction)))))
Geographical filter: India<br>Language filter: English	

Data collection and management

The results (titles and/or abstracts) of the search will be managed using Endnote (v. x7). Study selection will be performed on Endnote (v. x7). Data will be extracted on Microsoft Excel 2007.

Selection of studies

Studies will be reviewed based on the exclusion and inclusion criteria by two authors (SM and TL) independently in three stages. Stage one or title screening will include assessment of each title for inclusion in the review. If both authors reject a title, it will not be included in the review. Studies which are approved by either author will move to the second stage of appraisal. In the second or abstract screening stage will involve screening of abstracts of the titles selected in stage one for inclusion in the review. If both authors reject an abstract at this stage, it will not be included in the review. Studies which are approved by either author will move to the third and final stage of appraisal. Stage three, the full text screening stage, will comprise of screening the full text of the abstracts selected in stage two. Only those studies approved by both the authors at this stage will be included in the review. In the event of a study accepted by one author and rejected by another, a third author (MG) and a senior reviewer (SN or LL) will arbitrate and a consensus will be reached on whether to include the study or not.

For peer review only

For peer review only - http://bmjopen.bmj.com/site/about/guidelines.xhtml

Data extraction

The data extraction form was developed, using Microsoft Excel 2007, through an iterative process involving discussions and pilot testing. After a round of discussion among the authors, senior reviewers, subject and clinical experts, and statisticians, the form was pilot-tested on one study of each type to ensure that it adequately facilitated the collection of essential information required for the narrative synthesis. The key headings under which data extraction will be done include (1) Study Characteristics (2) Methodological characteristics (3) Treatment options and barriers to case management and (4) Other important information.

A final list of articles will be prepared for data extraction. This standardised, pre-tested data extraction form will be used independently by two authors (SM and TL) to extract data from the selected studies. Disagreements will be resolved in the presence of third (MG) and senior reviewer authors (SN and LL) by discussion and consensus. Any discrepancies regarding inclusion of the study in the review will be discussed with the team and advisory group, and a decision will be made regarding its inclusion in the review.

Dealing with missing data

In case of inadequacy, missing information, lack of clarity on information in methodology or if outcomes are missing, authors of the respective studies will be contacted in an attempt to obtain the required details. Despite this attempt, if the missing data retrieval on some aspects of the outcome (like clarity, inadequacy) is not possible, the study will be included in the narrative summary with a mention of the same.

Data Synthesis

A narrative summary will be performed to summarize the details of evidence. A discussion, where applicable, on study limitations that should be considered when interpreting the findings of the review will be included. The complete results of any analyses conducted, including the final search strategy, will be reported. No quality assessment of the included studies has been planned.

Reporting of the scoping **review:** A Preferred Reporting Items for Systematic reviews and Meta-analysis (PRISMA) chart will be created, to outline and summarise this study selection

For peer review only - http://bmjopen.bmj.com/site/about/guidelines.xhtml
For peer review only

process.[10] The findings of this review will be reported in accordance with the PRISMA Guidelines.[10 11]

Ethics and dissemination:

Ethics: Since this is a review involving analysis of secondary data which is available in the public domain, and does not involve human participants, ethical approval was not required.

Dissemination: The findings of the study will be shared with all stakeholders of this research. Knowledge dissemination workshops will be conducted with relevant stakeholders to ultimately transfer the evidence tailored to the stakeholder (e.g. policy briefs, publications, information booklets, etc).

Acknowledgements

We would like to thank the following individuals for their continuous support and guidance during this process of protocol development: Dr. Manoj Das, Director Projects, The INCLEN Trust International, New Delhi; Dr Anju Sinha, Deputy Director General, Scientist 'E', Division of Child Health, Indian Council of Medical Research, New Delhi; Dr. K.K. Diwakar, Professor and Head, Department of Neonatology, Associate Dean, Malankara Orthodox Syrian Church Medical College, Kerala; Mrs. Ratheebhai V., Senior Librarian and Information Scientist, at Manipal School at Communication, Manipal University, Manipal; Dr. Ravinder M. Pandey, Professor and Head, Department of Biostatistics, All India Institute of Medical Sciences, New Delhi; Dr. B. Shantharam Baliga, Professor, Department of Paediatrics, Kasturba medical college, Mangalore, Karnataka, ; Dr. Shirish Darak, Senior researcher, PRAYAS, Pune, Maharashtra; Dr. Unnikrishnan B., Associate Dean and Professor, Department of Community Medicine, Kasturba Medical College, Mangalore. We also thank Public Health Evidence South Asia (PHESA) and Manipal University, Manipal for providing the necessary institutional and infrastructural support for the project. We would also like to thank The INCLEN Trust International, New Delhi, and The Bill and Melinda Gates Foundation for the financial support which made this project possible.

Contributorship statement

SN is the guarantor of the review. SN, BV AND LL conceived the research idea and reviewed the manuscript. SN and LL provided overall technical guidance. In addition, LL assisted in

For peer review only

For peer review only - http://bmjopen.bmj.com/site/about/guidelines.xhtml

developing search terms. SM, TL and MG designed the protocol, drafted the manuscript and developed and pilot tested the search strategies and data extraction form.

Funding: This project is supported by a grant from Bill and Melinda Gates Foundation (grant OPP1084307) to The INCLEN Trust International and sub-grant to Manipal University (subgrant INC2015GNT004). The views expressed through this project do not necessarily represent the views of Bill and Melinda Gates Foundation or The INCLEN Trust International or Manipal University.

Data sharing statement: All data supporting this study will be provided as supplementary material together with the manuscript of the study's final results.

Competing Interests: All authors have completed the ICMJE uniform disclosure form at www.icmje.org/coi_disclosure.pdf and declare: all authors had financial support (grants) from Bill and Melinda Gates Foundation (grant OPP1084307) to The INCLEN Trust International and sub-grant to Manipal University (subgrant INC2015GNT004)., during the conduct of the study and for the submitted work; no financial relationships with any organisations that might have an interest in the submitted work in the previous three years; no other relationships or activities that could appear to have influenced the submitted work.

References

1. UNICEF. Committing to Child Survival: A Promise Renewed, Progress Report 2015. 2015. *New York, USA: UNICEF* September 2015
2. Rudan I, Boschi-Pinto C, Biloglav Z, et al. Epidemiology and etiology of childhood pneumonia. *Bulletin of the World Health Organization* 2008;86(5):408-16B.
3. Reiterer F. Neonatal pneumonia. *Neonatal Bacterial Infection InTech* 2013
4. Ghimire M, Bhattacharya S, Narain J. Pneumonia in South-East Asia Region: Public health perspective. *The Indian journal of medical research* 2012;135(4):459.
5. Duke T. Neonatal pneumonia in developing countries. *Archives of Disease in Childhood-Fetal and Neonatal Edition* 2005;90(3):F211-FF19.
6. UNICEF. Integrated Global Action Plan for Prevention and Control of Pneumonia and Diarrhoea (GAPPD) 2013 [accessed 2016 20/1/2016]. Available from: http://www.who.int/
7. UNICEF, World Health Organization. Global action plan for prevention and control of pneumonia (GAPP). 2009
8. Graham SM, English M, Hazir T, et al. Challenges to improving case management of childhood pneumonia at health facilities in resource-limited settings. *Bulletin of the World Health Organization* 2008;86(5):349-55.
9. Engle WD, Jackson GL, Sendelbach DM, et al. Pneumonia in term neonates: laboratory studies and duration of antibiotic therapy. *Journal of Perinatology* 2003;23(5):372-77.

For peer review only - http://bmjopen.bmj.com/site/about/guidelines.xhtml

10. Liberati A, Altman DG, Tetzlaff J, et al. The PRISMA statement for reporting systematic reviews and meta-analyses of studies that evaluate health care interventions: explanation and elaboration. *Annals of internal medicine* 2009;151(4):W-65-W-94.
11. Moher D, Liberati A, Tetzlaff J, et al. Preferred reporting items for systematic reviews and meta-analyses: the PRISMA statement. *Annals of internal medicine* 2009;151(4):264-69.

For peer review only

For peer review only - http://bmjopen.bmj.com/site/about/guidelines.xhtml

BMJ Open

Treatment options and Barriers to Case Management of Neonatal Pneumonia in India: a protocol for a scoping review

Journal:	*BMJ Open*
Manuscript ID	bmjopen-2017-017617.R2
Article Type:	Protocol
Date Submitted by the Author:	14-Jul-2017
Complete List of Authors:	Sreekumaran Nair, N; Manipal University, biostats<br>Lewis, Leslie; Manipal University Kasturba Medical College Manipal, paediatrics<br>Murthy, Shruti; Manipal University, Public Health Evidence South Asia (PHESA)<br>Godinho, Myron ; Manipal University, Public Health Evidence South Asia (PHESA)<br>Lakiang, Theophilus; Manipal University<br>Venkatesh , Bhumika; Manipal University
**Primary Subject Heading**:	Infectious diseases
Secondary Subject Heading:	Infectious diseases, Paediatrics
Keywords:	Neonate, Pneumonia, Treatment, Case management, Barriers, scoping review

SCHOLARONE™
Manuscripts

For peer review only

Treatment options and Barriers to Case Management of Neonatal Pneumonia in India: a protocol for a scoping review

N. Sreekumaran Nair[1], Leslie Edward Lewis[2], Shruti Murthy [3*], Myron Godinho [3**], Theophilus Lakiang[3***], Bhumika T. Venkatesh[4]

[1] Director, Public Health Evidence South Asia (PHESA), & Professor of Biostatistics and Head, Department of Statistics, Manipal University, Manipal. sree.nair@manipal.edu, nsknairmanipal@gmail.com

[2] Professor and Head, Neonatology Unit, Department of Pediatrics, Kasturba Medical College, Manipal University, Manipal. leslie.lewis@manipal.edu

[3] Research Associate, Public Health Evidence South Asia (PHESA), Manipal University, Manipal;

*itzshruti@gmail.com

**myrongodinho@gmail.com

***theo.lakiang@gmail.com

[4]Assistant Professor, Public Health Evidence South Asia (PHESA), Manipal University, Manipal; meetbhumika123@gmail.com

Correspondence to:
Dr. N. Sreekumaran Nair
Director, Public Health Evidence South Asia (PHESA), Ground Floor, Manipal EDU Building, and
Professor of Biostatistics and Head, Department of Statistics,
Level 6, Health Science Library Building,
Manipal University,
Manipal-576104, Karnataka, India
Email address: sree.nair@manipal.edu, nsknairmanipal@gmail.com
Telephone number: +91-8202922407

PROSPERO 2016:CRD42016045449

For peer review only - http://bmjopen.bmj.com/site/about/guidelines.xhtml

Treatment options and Barriers to Case Management of Neonatal Pneumonia in India: a protocol for a scoping review

Abstract

Introduction: India contributes to the highest neonatal deaths globally. Case management is said to be the cornerstone of pneumonia control. Much of the published evidence focuses on children aged 1 to 59 months. This scoping review, thus, aims to identify the treatment options for and barriers to case management of neonatal pneumonia in India.

Methods and Analysis: This protocol is part of a series of three reviews on neonatal pneumonia in India. Studies addressing treatment of or barriers to case management of neonatal pneumonia in Indian context, published in English in peer-reviewed and indexed journals will be eligible for inclusion. Electronic search will be conducted on nine databases. Hand searching and snowballing will be done for published and grey literature will be performed. Selection of studies will be done in title, abstract and full text stages. A narrative summary will be performed to summarize the details of evidence.

Ethics and dissemination: Since this is a review involving analysis of secondary data which is available in the public domain, and does not involve human participants, ethical approval was not required. The findings of the study will be shared with all stakeholders of this research. Knowledge dissemination workshops will be conducted with relevant stakeholders to ultimately transfer the evidence tailored to the stakeholder (e.g. policy briefs, publications, information booklets, etc).

Keywords: Neonate, Pneumonia, Treatment, Case management, Barriers, Scoping Review, India

For peer review only

Strengths and limitations of the study

- Most documented literature in pneumonia case management address post-neonatal age groups. First review to consolidate research on the treatment modalities, their implementation, and the barriers to the case management of neonatal pneumonia in India.

- A comprehensive search strategy was developed over 9 databases including relevant regional databases and grey literature published in any year.

- The review will narratively summarize published literature. No quality assessment of included studies is planned.

Introduction

Globally, 5.9 million children died in 2015, out of which 2.6 million were neonates.[1] India accounts for more neonatal deaths than any other country.[2] More than half of the child deaths from pneumonia occur during the newborn period.[3] In India, economic deprivation, impaired access to healthcare, harmful child-rearing practices, malnutrition and indoor air pollution are all major risk factors for pneumonia.[4 5] Neonatal pneumonia is particularly difficult to define and classify, as witnessed by differing definitions in different studies.[3 6]

In 2008, the World Health Organization (WHO)- United Nations Children's Fund (UNICEF) formulated the Global Action plan for the Prevention and control of Pneumonia (GAPP),[7] and in India, the Integrated Management of Neonatal & Childhood Illness (IMNCI) is now an important part of the national strategy to control childhood illness. Timely detection, effective case management, and prompt referral can reduce child morbidity and mortality due to pneumonia.[8] However, this is challenging in regions where co-morbid conditions (e.g. tuberculosis, malaria, AIDS) and antibiotic resistance prevail.[6] These difficulties are compounded by the clinical overlap of neonatal sepsis and pneumonia,[3] and obscured by conditions like hyaline membrane disease which mimic neonatal pneumonia, and impede detection in the absence of bacteriological confirmation.[5] Moreover, injudicious antibiotic therapy could lead to resistance or treatment failure.[9] A multitude of factors such as these pose special challenges to the initiation and maintenance of treatment, resulting in excessive morbidity and mortality.[8] However, most documented difficulties in pneumonia

For peer review only - http://bmjopen.bmj.com/site/about/guidelines.xhtml

case management address post-neonatal age groups, and fail to discuss neonatal treatment issues.

There is recognized need for consolidated research on the treatment modalities, their implementation, and the barriers to the case management of neonatal pneumonia in India. This evidence is required to inform the development of interventions, education and preventive strategies to combat this scourge of India's newborn. Thus this scoping review will attempt to synthesize evidence on different treatment options existing for neonatal pneumonia and the factors hindering effective case management of neonatal pneumonia in the Indian context.

The objectives of this scoping review are to identify the

1. Treatment options for neonatal pneumonia, and
2. Barriers to case management of neonatal pneumonia in the Indian context.

This protocol is part of a larger mixed-methods research project consisting of a qualitative study and a trilogy of two systematic and one scoping reviews on neonatal pneumonia in India addressing risk factors, management and predictors of mortality due to neonatal pneumonia in the Indian context.

Methods and analysis

This review will be conducted from August 2016 to October 2017.

Criteria for considering studies for this review

Types of studies

Inclusion criteria: Studies eligible for inclusion should have been conducted among neonates with pneumonia (or sepsis) in the Indian context, and their stakeholders. Primary studies, (of any study design including editorials, case reports, case series, cross-sectional studies, case control studies, cohort studies, intervention studies and qualitative studies), policy papers, guidelines, reports, and fact sheets, addressing treatment of or barriers to case management of neonatal pneumonia in Indian context were eligible to be included in the review. Studies have to be published in English language in indexed and peer-reviewed journals to be eligible for inclusion.

For peer review only - http://bmjopen.bmj.com/site/about/guidelines.xhtml

Exclusion criteria: The following studies will be excluded: all types of reviews, meta-analysis, conference papers and reports which do not report on treatment or barriers to case management of neonatal pneumonia in an Indian context.

Operational definitions: For the purpose of this review, treatment was operationally defined as ‘any specific or supportive treatment administered to a neonate with pneumonia’; case management was defined as ‘detection, investigation, treatment, referral, monitoring, support or follow up of pneumonia in a neonate either in the facility or community’[8]; and barrier to case management was defined as ‘any difficulty or obstacle during the case management of neonatal pneumonia’. Though we defined these terms in the beginning, our intention will also be to capture the definitions, where available, as reported by the authors and present them in the narrative synthesis.

Type of participants: Neonates with pneumonia in Indian context.

Outcomes of this review: Outcomes of this review will be (a) specific and supportive treatment of- and (b) barriers to case management of neonatal pneumonia in Indian context.

Search methods for identification of studies:

A comprehensive and relevant search strategy to identify all relevant studies will be developed by reviewing literature and discussion with subject experts and an information scientist. The search terms used and search strategies for PubMed have been provided in Tables 1 and 2.

Electronic searches: We will search PubMed, Ovid Medline, ProQuest, EMBASE, CINAHL, Web of Science, SCOPUS, WHOLIS and IndMED.

Hand searching: Hand searching will be conducted for reports/ guidelines/ journal volumes not included in electronic databases and conference proceedings to review the references and contact the authors for full text of identified literature.

Searching the grey literature: Potential sources of grey literature will include Shodhganga (INFLIBNET) and Government of India databases for reports, fact sheets and guidelines/ policies in the Indian context.

For peer review only - http://bmjopen.bmj.com/site/about/guidelines.xhtml

Reference lists: Snowballing will be conducted to screen the references of identified literature for potentially relevant studies.

Table 1: Search strategy for treatment options (PubMed):

Strategy: #1 AND #2 AND #3	
#1	(((Neonate* OR childhood OR neonatal* OR newborn* OR "young infant" OR child OR paediatric OR pediatric* OR "neonatal period" OR infant* OR "newborn infant")))
#2	((((((((((((((((((((((((((((((Pneumonia*) OR Pneumon*) OR "community acquired pneumonia") OR "congenital pneumonia") OR "hospital acquired pneumonia") OR "nosocomial pneumonia") OR "ventilator associated pneumonia") OR "early onset pneumonia") OR "late onset pneumonia") OR "infective pneumonia") OR "infectious pneumonia") OR "meconium aspiration syndrome") OR "meconium aspiration") OR "lipoid pneumonia") OR sepsis*) OR "acute respiratory infections") OR "early onset sepsis") OR "chemical pneumonia") OR "aspiration pneumonia") OR "late onset sepsis") OR infection*) OR "nosocomial infection") OR "early onset infection") OR "late onset infection") OR "acute lower respiratory infection") OR "hospital acquired infection") OR "congenital infection") OR "viral pneumonia") OR "gastro esophageal reflux disease") OR "cystic fibrosis")
#3	(((Treatment* OR Therap* OR "Patient care management" OR "Case management programs" OR "Home based neonatal care" OR "Case Management" OR "Clinical case management" OR "Community case management" OR "Integrated community case management" OR "Home based newborn care" OR "Case management models" OR Antibiotic* OR Ventilation* OR "Intensive care units" OR "Intensive care" OR "Neonatal intensive care units" OR "Special Newborn Care Units" OR "Injectable antibiotic" OR "oral antibiotic" OR "supportive therapy" OR "specific therapy" OR "specific treatment" OR "Supportive treatment"))))
Geographical filter: India<br>Language Filter: English	

For peer review only

Table 2: Search strategy for barriers to case management (PubMED)

Strategy: #1 AND #2 AND #3 AND #4	
#1	((((Neonate* OR childhood OR neonatal* OR newborn* OR "young infant" OR child OR paediatric OR pediatric* OR "neonatal period" OR infant* OR "newborn infant")))
#2	((((((((((((((((((((((((((((((Pneumonia*) OR Pneumon*) OR "community acquired pneumonia") OR "congenital pneumonia") OR "hospital acquired pneumonia") OR "nosocomial pneumonia") OR "ventilator associated pneumonia") OR "early onset pneumonia") OR "late onset pneumonia") OR "infective pneumonia") OR "infectious pneumonia") OR "meconium aspiration syndrome") OR "meconium aspiration") OR "lipoid pneumonia") OR sepsis*) OR "acute respiratory infections") OR "early onset sepsis") OR "chemical pneumonia") OR "aspiration pneumonia") OR "late onset sepsis") OR infection*) OR "nosocomial infection") OR "early onset infection") OR "late onset infection") OR "acute lower respiratory infection") OR "hospital acquired infection") OR

For peer review only - http://bmjopen.bmj.com/site/about/guidelines.xhtml

	"congenital infection") OR "viral pneumonia") OR "gastro esophageal reflux disease") OR "cystic fibrosis")
#3	((Treatment* OR Therap* OR "Patient care management" OR "Case management programs" OR "Home based neonatal care" OR "Case Management" OR "Clinical case management" OR "Community case management" OR "Integrated community case management" OR "Home based newborn care" OR "Case management models" OR Antibiotic* OR Ventilation* OR "Intensive care units" OR "Intensive care" OR "Neonatal intensive care units" OR "Special Newborn Care Units" OR "Injectable antibiotic" OR "oral antibiotic" OR "supportive therapy" OR "specific therapy" OR "specific treatment" OR "Supportive treatment"))
#4	(((Barriers* OR challenging OR challenge* OR obstacle* OR difficult* OR drawback OR problem* OR hurdle* OR hindrance* OR hinder* OR gap* OR cost* OR utilization OR satisfaction)))))
Geographical filter: India<br>Language filter: English	

Data collection and management

The results (titles and/or abstracts) of the search will be managed using Endnote (v. x7). Study selection will be performed on Endnote (v. x7). Data will be extracted on Microsoft Excel 2007.

Selection of studies

Studies will be reviewed based on the exclusion and inclusion criteria by two authors (SM and TL) independently in three stages. Stage one or title screening will include assessment of each title for inclusion in the review. If both authors reject a title, it will not be included in the review. Studies which are approved by either author will move to the second stage of appraisal. In the second or abstract screening stage will involve screening of abstracts of the titles selected in stage one for inclusion in the review. If both authors reject an abstract at this stage, it will not be included in the review. Studies which are approved by either author will move to the third and final stage of appraisal. Stage three, the full text screening stage, will comprise of screening the full text of the abstracts selected in stage two. Only those studies approved by both the authors at this stage will be included in the review. In the event of a study accepted by one author and rejected by another, a third author (MG) and a senior reviewer (SN or LL) will arbitrate and a consensus will be reached on whether to include the study or not.

For peer review only

Data extraction and Charting the results

A charting form was developed, in Microsoft Excel 2007, through an iterative process involving discussions and pilot testing. After a round of discussion among the authors, senior reviewers, subject and clinical experts, and statisticians, the form was pilot-tested on one study of each type to ensure that it adequately facilitated the collection of essential information required for the narrative synthesis. The key headings under which charting will be done include (1) Study Characteristics (2) Methodological characteristics (3) Treatment options and barriers to case management and (4) Other important information.

This standardised, pre-tested charting form will be used independently by two authors (SM and TL) to extract data from the selected studies. Disagreements will be resolved in the presence of third (MG) and senior reviewer authors (SN and LL) by discussion and consensus. Any discrepancies regarding inclusion of the study in the review will be discussed with the team and advisory group, and a decision will be made regarding its inclusion in the review.

Dealing with missing data

In case of inadequacy, missing information, lack of clarity on information in methodology or if outcomes are missing, authors of the respective studies will be contacted in an attempt to obtain the required details. Despite this attempt, if the missing data retrieval on some aspects of the outcome (like clarity, inadequacy) is not possible, the study will be included in the narrative summary with a mention of the same.

Reporting the results

The complete results of any analyses conducted, including the final search strategy, will be reported. Results will be in tabular form supplemented with a descriptive summary of the findings. Tables will present the characteristics of included studies (study ID, year of publication, location and setting, study design and sample size, definitions adopted in the studies, treatments recommended by guidelines, treatments reported by primary research studies for neonatal pneumonia, and barriers reported during the case management of neonatal pneumonia. The descriptive summary will include details about the study objectives, the approach adopted and the findings. A discussion, where applicable, on study

For peer review only - http://bmjopen.bmj.com/site/about/guidelines.xhtml

limitations that should be considered when interpreting the findings of the review will be included. No quality assessment of the included studies has been planned.

A Preferred Reporting Items for Systematic reviews and Meta-analysis (PRISMA) chart will be created, to outline and summarise this study selection process.[10] The findings of this review will be reported in accordance with the "Guidance for conducting systematic scoping reviews".[11]

Ethics and dissemination:

Ethics: Since this is a review involving analysis of secondary data which is available in the public domain, and does not involve human participants, ethical approval was not required.

Dissemination: The findings of the study will be shared with all stakeholders of this research. Knowledge dissemination workshops will be conducted with relevant stakeholders to ultimately transfer the evidence tailored to the stakeholder (e.g. policy briefs, publications, information booklets, etc).

Acknowledgements

We would like to thank the following individuals for their continuous support and guidance during this process of protocol development: Dr. Manoj Das, Director Projects, The INCLEN Trust International, New Delhi; Dr Anju Sinha, Deputy Director General, Scientist 'E', Division of Child Health, Indian Council of Medical Research, New Delhi; Dr. K.K. Diwakar, Professor and Head, Department of Neonatology, Associate Dean, Malankara Orthodox Syrian Church Medical College, Kerala; Mrs. Ratheebhai V., Senior Librarian and Information Scientist, at Manipal School at Communication, Manipal University, Manipal; Dr. Ravinder M. Pandey, Professor and Head, Department of Biostatistics, All India Institute of Medical Sciences, New Delhi; Dr. B. Shantharam Baliga, Professor, Department of Paediatrics, Kasturba medical college, Mangalore, Karnataka, ; Dr. Shirish Darak, Senior researcher, PRAYAS, Pune, Maharashtra; Dr. Unnikrishnan B., Associate Dean and Professor, Department of Community Medicine, Kasturba Medical College, Mangalore. We also thank Public Health Evidence South Asia (PHESA) and Manipal University, Manipal for providing the necessary institutional and infrastructural support for the project. We would also like to thank The INCLEN Trust

For peer review only

For peer review only - http://bmjopen.bmj.com/site/about/guidelines.xhtml

International, New Delhi, and The Bill and Melinda Gates Foundation for the financial support which made this project possible.

Contributorship statement

SN is the guarantor of the review. SN, BV AND LL conceived the research idea and reviewed the manuscript. SN and LL provided overall technical guidance. In addition, LL assisted in developing search terms. SM, TL and MG designed the protocol, drafted the manuscript and developed and pilot tested the search strategies and data extraction form.

Funding: This project is supported by a grant from Bill and Melinda Gates Foundation (grant OPP1084307) to The INCLEN Trust International and sub-grant to Manipal University (subgrant INC2015GNT004). The views expressed through this project do not necessarily represent the views of Bill and Melinda Gates Foundation or The INCLEN Trust International or Manipal University.

Data sharing statement: All data supporting this study will be provided as supplementary material together with the manuscript of the study's final results.

Competing Interests: All authors have completed the ICMJE uniform disclosure form at www.icmje.org/coi_disclosure.pdf and declare: all authors had financial support (grants) from Bill and Melinda Gates Foundation (grant OPP1084307) to The INCLEN Trust International and sub-grant to Manipal University (subgrant INC2015GNT004)., during the conduct of the study and for the submitted work; no financial relationships with any organisations that might have an interest in the submitted work in the previous three years; no other relationships or activities that could appear to have influenced the submitted work.

References

1. UNICEF. Committing to Child Survival: A Promise Renewed, Progress Report 2015. 2015. *New York, USA: UNICEF* September 2015
2. Rudan I, Boschi-Pinto C, Biloglav Z, et al. Epidemiology and etiology of childhood pneumonia. *Bulletin of the World Health Organization* 2008;86(5):408-16B.
3. Reiterer F. Neonatal pneumonia. *Neonatal Bacterial Infection InTech* 2013
4. Ghimire M, Bhattacharya S, Narain J. Pneumonia in South-East Asia Region: Public health perspective. *The Indian journal of medical research* 2012;135(4):459.
5. Duke T. Neonatal pneumonia in developing countries. *Archives of Disease in Childhood-Fetal and Neonatal Edition* 2005;90(3):F211-FF19.

For peer review only - http://bmjopen.bmj.com/site/about/guidelines.xhtml

6. UNICEF. Integrated Global Action Plan for Prevention and Control of Pneumonia and Diarrhoea (GAPPD) 2013 [cited 2016 20/1/2016]. Available from: http://www.who.int/maternal_child_adolescent/documents/global_action_plan_pneumonia_diarrhoea/en.
7. UNICEF, Organization WH. Global action plan for prevention and control of pneumonia (GAPP). 2009
8. Graham SM, English M, Hazir T, et al. Challenges to improving case management of childhood pneumonia at health facilities in resource-limited settings. *Bulletin of the World Health Organization* 2008;86(5):349-55.
9. Engle WD, Jackson GL, Sendelbach DM, et al. Pneumonia in term neonates: laboratory studies and duration of antibiotic therapy. *Journal of Perinatology* 2003;23(5):372-77.
10. Liberati A, Altman DG, Tetzlaff J, et al. The PRISMA statement for reporting systematic reviews and meta-analyses of studies that evaluate health care interventions: explanation and elaboration. *Annals of internal medicine* 2009;151(4):W-65-W-94.
11. Peters MD, Godfrey CM, Khalil H, et al. Guidance for conducting systematic scoping reviews. *International journal of evidence-based healthcare* 2015;13(3):141-46.

For peer review only

For peer review only - http://bmjopen.bmj.com/site/about/guidelines.xhtml
